# Generalized Additive Mixed-Models for Pharmacology Using Integrated Discrete Multiple Organ Co-Culture

**DOI:** 10.1371/journal.pone.0152985

**Published:** 2016-04-25

**Authors:** Thomas Ingersoll, Stephanie Cole, Janna Madren-Whalley, Lamont Booker, Russell Dorsey, Albert Li, Harry Salem

**Affiliations:** 1 Defense Threat Reduction Agency, Joint CBRN Center of Excellence, Aberdeen Proving Ground, Maryland, United States of America; 2 US Army Research, Development, and Engineering Command, Edgewood Chemical Biological Center, Aberdeen Proving Ground, Maryland, United States of America; 3 Food and Drug Administration, Silver Spring, Maryland, United States of America; 4 In Vitro ADMET Laboratories, Columbia, Maryland, United States of America; National Institute of Environmental and Health Sciences, UNITED STATES

## Abstract

Integrated Discrete Multiple Organ Co-culture (IDMOC) is emerging as an in-vitro alternative to in-vivo animal models for pharmacology studies. IDMOC allows dose-response relationships to be investigated at the tissue and organoid levels, yet, these relationships often exhibit responses that are far more complex than the binary responses often measured in whole animals. To accommodate departure from binary endpoints, IDMOC requires an expansion of analytic techniques beyond simple linear probit and logistic models familiar in toxicology. IDMOC dose-responses may be measured at continuous scales, exhibit significant non-linearity such as local maxima or minima, and may include non-independent measures. Generalized additive mixed-modeling (GAMM) provides an alternative description of dose-response that relaxes assumptions of independence and linearity. We compared GAMMs to traditional linear models for describing dose-response in IDMOC pharmacology studies.

## Introduction

A goal of pharmacological studies is to predict the dose-response relationship of a chemical in humans and any potential toxicological effects [1, 2, 3, 4]. A common approach is to employ *in vivo* animal testing following the assumption that animals have evolved complex functional organ systems similar to those of humans and therefore should be useful predictors of a given human response. However, failure of animal models to accurately predict a response in humans for many compounds, cost, and ethical concerns associated with the use of animal resources are limiting factors to the utility of animal models [4]. Thus, incentive is mounting to develop alternatives.

*In vitro* cell culture systems, may offer a viable alternative to the use of animals for many pharmacological tests. *In vitro* testing has advantages over *in vivo* testing, including lower cost, and higher throughput. Another important difference between *in vitro* and *in vivo* systems is that the end-points are more mechanistic like apoptosis as opposed to the more apical *in vivo* endpoints such as animal lethality. This has important consequences for mathematical models describing *in vitro* dose-response.

Generalized linear models (GLM) for dose-response *in vivo*, such as probit-link models, are familiar to many pharmacologists [5, 6]. GLM’s simplify dose range interpretation in animal models. Further, GLMs relax normality assumptions inherent in linear models so that non-normal data such binary lethality may be accommodated [7]. Yet, end-points for in vitro studies are not restricted to binary responses, and at the same time may exhibit internal extrema to which probit-link linear models are insensitive.

*In vitro*, a proxy of lethality or a physiological mechanism is measured. For example, fluorescent dyes can be used to measure changes in enzyme activity or cell membrane integrity. *In vitro* responses are often measured on continuous, rather than binomial scales and are typically non-linear [1]. Applying non-linear regression models as opposed to traditional GLM should reduce error. While differences in response associated with increased dose can be called a *trend* in a linear system, trend changes continuously in a non-linear system. Therefore we define the *dose response trajectory* as changes in response in the non-linear system [8]. As such, a trajectory can display internal extrema, such as a maximum dose-response, to which linear dose-response trends are insensitive.

Non-linear models are rapidly gaining acceptance in the toxicology community, and are supported by advances in software such as the Environmental Protection Agency’s BDMS [3], which contains a selection of non-linear models. However, the particular form of a non-linear dose response isn’t always known a-priori, and coercing a model into a preselected curvature can result in poor model fit. Analytic problems, such as heteroscedasticity, excessively broad confidence intervals, and misestimates of intercept values can occur when GLMs or non-linear models with coerced curvature are applied. Misestimated intercept values can be particularly problematic when seeking to isolate the effects of a toxicant from background levels. While simple curved trajectories, such as the parabolic flight of a projectile through a gravitational field may be easily quantified based on well-understood physical laws, we found that *in-vitro* dose-response trajectories often assumed shapes of complex curvature due to the interaction of biological effects, some of which were not well-understood, and could not be quantified a-priori.

Generalized additive models (GAMs) have the advantage of objective curvature selection, where data, rather than the researcher’s a-priori conception, determine the shape of the model [9, 10]. Generalized additive mixed-models (GAMMs) have the additional advantage of relaxed independence assumptions [11, 12], accommodating the repeated-measures experimental design often found in *in vitro* toxicology studies. GAMMs can eliminate pseudo-replication, improve model fit, increase reliability of confidence intervals, and provide better local estimates of dose response and intercepts than other models.

Mathematical models of dose response provide a more generalized, simplified, and interpretable description of dose response compared to less formal summaries of data such as bar-graphs. Models allow statistics such as the 50% lethal response (LD_50_), intercept values, and standard measures of uncertainty such as confidence intervals to be estimated. Furthermore, mathematical models can be transformed or scaled, so that their predictions more closely resemble our expectations about *in vivo* systems. As such, mathematical representations of *in vitro* models may better allow us to indirectly observe processes and predict patterns we would expect to see with *in vivo* systems, if data on these latter systems were available.

Akaike’s information criterion (AIC) is a method for selecting among candidate models based on a comparison of model likelihoods calculated from experimental data [13]. The process is similar to using likelihood ratio tests (LRT) for model comparison except that AIC includes a penalty for model complexity and compares models by relative informational weight, rather than using tests of null hypotheses. Each competing model is allocated a proportional model weight (*w*_*i*_) out of a total weight of one for all models compared. The result is that all candidate models may be directly compared, so are not restricted to the nested-pairwise comparisons of LRT [14]. AIC selects the most parsimonious model or group of models for model-based inference, rather than using formal tests of null hypotheses [15]. Model confidence intervals are then estimated in lieu of p-values.

In the current study we report on the validation of mathematical models in the analysis of *in vitro* studies for metabolic toxicity using Integrated Discrete Multiple Organ Co-culture (IdMOC). IdMOC further enhances simulation of *in vivo* systems because it allows the study of interactions between tissues derived from separate progenitor cell lines [16, 17]. For example, cells with a predominately metabolic role, such as hepatocytes, may be cultured alongside connective tissue-derived cells, such as fibroblasts [17]. IdMOC allows interaction between the disparate cell types by connection through a shared liquid medium which, in turn, permits transport of soluble metabolites [17]. That is, metabolites produced in one cell type are free to diffuse to the other. This method of co-culture creates conditions for toxicology research that could be expected to more closely resemble those of *in vivo* systems, when compared to cultures of single cell lines [16]. Because of the complexity of interacting cell types, substantial deviation from linear dose response should be expected in IdMOC systems.

## Methods

### Cell Culture, Staining, and High Content Analysis

Cell culture, toxicant exposure, and staining were conducted essentially as described in literature [17]. Briefly, 7,000 3T3-L1 cells (ATCC, Manassas, VA) per well were cultured in the presence and absence of 35,000 cryopreserved human hepatocytes per well (lot #HH1020, In Vitro ADMET Laboratories, Columbia, MD) in collagen-coated 96 well IdMOC plates (In Vitro ADMET Laboratories, Columbia, MD). In these experiments, 3 of the 6 inner wells in the IdMOC chambers contained 3T3-L1 cells, while the remaining 3 contained either hepatocytes (co-culture) or Universal Primary Cell Plating Medium (mono-culture; In Vitro ADMET Laboratories, Columbia, MD). Cells were dispensed into wells of the IdMOC plate and allowed to attach to the substrate for 4 hours in a 37°C humidified incubator with 5% CO_2_, after which they were exposed to toxicant diluted in Hepatocyte Induction Medium (HIM; In Vitro ADMET Laboratories, Columbia, MD). Cyclophosphamide was dissolved directly into HIM, while stocks of 4-aminophenol and ticlopidine were first dissolved in dimethyl sulfoxide (DMSO) and paraoxon was dissolved in ethanol before dilution in HIM. The DMSO concentration was maintained at 0.25% and 0.33% in all wells for the 4-aminophenol and ticlopidine exposures, respectively, while the ethanol concentration was maintained at 2.5% in all wells for the paraoxon exposure. The cells were exposed to 1.2 mL per chamber of toxicant diluted in HIM for 24 hours in a humidified incubator with 5% CO_2_ at 37°C before staining.

After 24 hours, the toxicant was removed from the IdMOC chambers and was replaced with phosphate-buffered saline containing 1 μM calcein AM (live stain) and 2 μM Hoechst 33342 (nuclear stain). The cells were incubated with stain for 1 hour at room temperature before high content analysis was conducted.

The Target Activation BioApplication of a Cellomics Arrayscan VTI HCS Reader (ThermoFisher, Pittburgh, PA) was employed for high content analysis. Two channels, XF93-Hoechst (nuclear stain) and XF93-FITC (live stain), under 10x magnification were used to identify cells (nuclear stain) and measure the fluorescence intensity of the live stain. Five hundred cells per well were analyzed, and the mean total intensity of the live stain fluorescence was reported in the vHCS:ViewTM software, from which the data were exported and analyzed further in Microsoft Excel 2007 (Microsoft Corporation, Redmond, WA). The fluorescence intensity of the experimental wells was scaled to that of the vehicle control wells.

### Modeling and Analysis

Models were produced using computational software R version 3.01 [18], and the R library *mgcv* [10].

For each endpoint response of the fibroblasts for each toxicant, the following models were compared:

$$E\left[y\right]=u_{jk}+s_{1}\left(Conc\times Co\right)\;\left(\text{GAMM with interactions}\right)$$ (1)

$$E\left[y\right]=u_{jk}+s_{1}\left(Conc\right)+\beta_{1}\left(Co\right)\;\left(\text{Main effects GAMM}\right)$$ (2)

$$E\left[y\right]=u_{jk}+\beta_{1}\left(Conc\right)+\beta_{2}\left(Co\right)+\beta_{3}\left(Conc\times Co\right)\;\left(\text{ANCOVA}\right)$$ (3)

$$E\left[y\right]=u_{jk}+\beta_{1}\left(Co\right)\;\left(\text{ANOVA}\right)$$ (4)

$$E\left[y\right]=u_{jk}+\mu \left(y\right)\;\left(\text{Intercept}\right)$$ (5)

Where *E*[*y*] was the expected florescence, *u*_*jk*_ was a random intercept for well *j* in plate *k*, *s*_1_ was a smoothing function (here, cubic regression splines), *β*_1:3_ were coefficients associated with each linear term, *Conc* was the concentration of each agent, and *Co* was the co-cultured vs. mono-culture category.

Non-independence in preparation heterogeneity was controlled using the random intercept terms [12]. A Gaussian distribution was assumed for all dependent variables.

AIC was used to select models for inference, and weights were tabulated for model comparisons across all candidates (Table 1). First, the saturated model (GAMM with interactions; Eq 1), was used to select random terms using AIC [12]. Second, major model classes given by Eqs 1–5 were compared using AIC to select the level of inference supported by the data (Table 1a–1d). Following initial comparisons, a step-wise method of model reduction was applied, where appropriate, to determine if further simplification was warranted (Table 2a and 2b). AIC weights [15] were calculated to compare random-effects terms, and to compare fixed-effects.

**Table 1:** AIC comparison of linear and non-linear dose-response models.

A) 4-aminophenol				
Model	Terms	AIC	ΔAIC	Wi
s(Conc) + Co | Well	6	1123.30	0.00	1.00
s(Conc X Co) | Well	7	1140.00	16.70	0.00
Conc + Co + Conc X Co | Well	6	1166.17	42.87	0.00
Co | Well	4	1203.08	79.78	0.00
1| Well	3	1205.14	81.84	0.00
B) Cyclophosphamide				
Model	Terms	AIC	ΔAIC	Wi
s(Conc X Co) | Well	7	1329.21	0.00	0.95
Conc + Co + Conc X Co | Well	6	1335.12	5.91	0.05
s(Conc) + Co | Well	6	1354.21	25.00	0.00
1| Well	3	1360.16	30.95	0.00
Co | Well	4	1361.80	32.59	0.00
C) Paroxon				
Model	Terms	AIC	ΔAIC	Wi
s(Conc) + Co | Well	6	1133.15	0.00	1.00
s(Conc X Co) | Well	7	1172.42	39.27	0.00
Conc + Co + Conc X Co | Well	6	1245.78	112.63	0.00
1| Well	3	1260.07	126.92	0.00
Co | Well	4	1262.04	128.88	0.00
D) Ticlopedine				
Model	Terms	AIC	ΔAIC	Wi
s(Conc) + Co | Well	6	1227.34	0.00	0.71
Conc + Co + Conc X Co | Well	6	1229.79	2.45	0.21
s(Conc X Co) | Well	7	1232.95	5.62	0.04
Co | Well	4	1233.47	6.14	0.03
1| Well	3	1239.60	12.27	0.00

**Table 2:** AIC comparison for simplification of indicated models.

A) 4-aminophenol				
Model	Terms	AIC	ΔAIC	Wi
s(Conc) + Co | Well	6	1123.30	0.00	1.00
s(Conc) | Well	5	1146.30	23.00	0.00
Conc | Well	4	1171.35	48.05	0.00
B) Paroxon				
Model	Terms	AIC	ΔAIC	Wi
s(Conc) | Well	5	1131.96	0.00	0.64
s(Conc) + Co | Well	6	1133.15	1.19	0.36
Conc | Well	4	1241.83	109.87	0.00
C) Ticlopedine				
Model	Terms	AIC	ΔAIC	Wi
s(Conc) + Co | Well	6	1227.34	0.00	0.99
s(Conc) | Well	5	1237.10	9.77	0.01
Conc | Well	4	1240.86	13.52	0.00

AIC preferred models were rendered graphically for interpretation, along with selected graphic comparisons between competing models. Graphs were produced by predicting response values and confidence intervals across a range of fixed concentration values, using the R function *predict* [19].

For illustrative purposes, GAMMs compared to ANCOVA and ANOVA, and response to cyclophosphamide were also weighted and graphed. Pearson residuals for the 3 models were compared using a LOESS smoother with span of 0.5 [12].

## Results

### Toxicant Selection

The categories of toxicants chosen in the experiments described herein are as follows: hepatotoxic (ticlopidine), generally cytotoxic (paraoxon), activated by hepatocytes (cyclophosphamide), and detoxified by hepatocytes (4-aminophenol). Ticlopedine is an anti-platelet drug that has been shown to induce hepatotoxicity both *in vivo* [20] and *in vitro* [21], while it is expected to be less toxic to non-hepatic cells. Paraoxon is an organophosphate insecticide that is generally toxic to a variety of cell types [22, 23], and as such is expected to induce cytotoxicity in both hepatocytes and 3T3-L1 cells in our cell culture model. Cyclophosphamide is a chemotherapeutic agent that is known to require metabolic activation by hepatocytes in order to form the cytotoxic metabolites 4-hydroxycyclophosphamide and phosphoramide mustard [24, 25]. In previous IdMOC experiments, it has been shown that cyclophosphamide is more toxic to 3T3-L1 cells that are cultured in the presence of hepatocytes compared to 3T3-L1 cells in monoculture [16]. The toxic industrial chemical 4-aminophenol is known to be detoxified by hepatocytes [26, 27], and it is expected that this compound will be more toxic to 3T3-L1 cells grown in monoculture compared to those grown in the presence of hepatocytes in IdMOC plates.

### Model-selection

For cyclophosphamide and ticlopedine, preferred models included full interactions between smoothed concentration and culture category (co-culture vs. monoculture). However, for ticlopedine, model preference was weak, with a model weight (*w*_*i*_) of only 0.52 for the model with interactions. Preferred models for 4-aminophenol and paroxon included a smoothed term for concentration. Model preference for smoothed concentration only in paroxon, with no term for co-culture category, was somewhat weak, with a weight 0.64 over a weight of 0.36 for a model including the linear category term. The preferred model for 4-aminophenol included a linear term for co-culture category, with no interaction between co-culture category and concentration, smoothed or linear.

### Graphics

**Fig 1:**
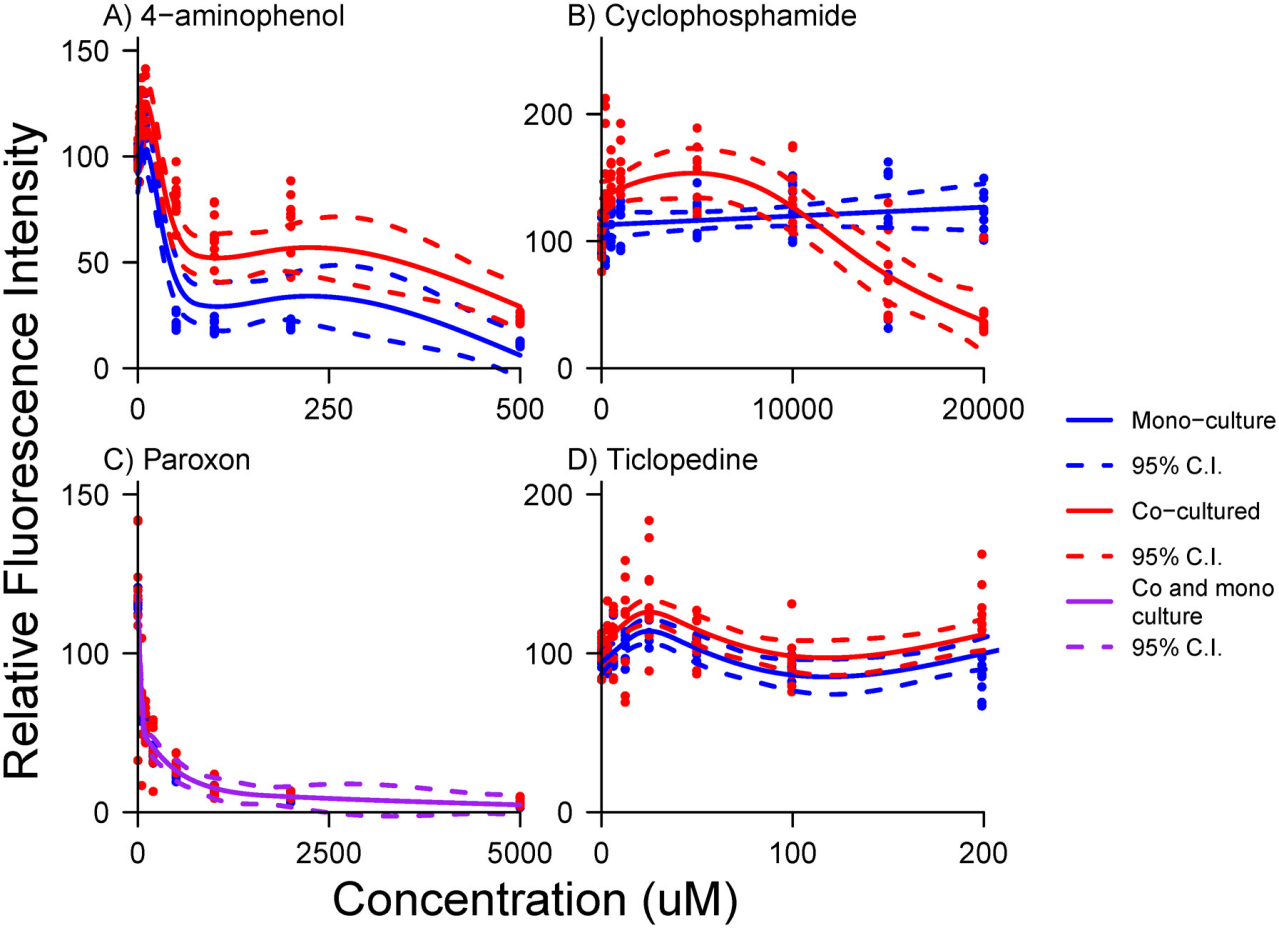
Comparison of mono and co-cultured dose-response trajectories in AIC-selected models for 4 compounds. A) 4−aminophenol exhibited separate intercepts but equivalent slopes for mono-cultured fibroblasts (blue trace) and fibroblasts co-cultured with hepatocytes (red trace). B) Cyclophosphamide exhibited separate trajectories for mono-cultured fibroblasts (blue trace) and fibroblasts co-cultured with hepatocytes (red trace). C) Paroxon exhibited indistinguishable trajectories for mono-cultured fibroblasts and fibroblasts co-cultured with hepatocytes (purple trace). D) Ticlopedine exhibited separate intercepts but equivalent slopes for mono-cultured fibroblasts (blue trace) and fibroblasts co-cultured with hepatocytes (red trace).

Cyclophosphamide and ticlopedine exhibited separate trajectories for co-cultured and monocultured fibroblasts (Fig 1). This indicates that the effect of increasing dose is different in co-cultured and monoculture for these toxicants. For both toxicants, little toxic effect was seen in monocultured fibroblasts, with approximately linear and level response to increasing concentrations. Co-cultured fibroblasts exhibited biphasic response, changing from a compensatory response to a toxic response to cyclophosphamide at high concentrations. Co-culture and monoculture exhibited identical trajectory patterns in both 4-aminophenol and paroxon, with increasing toxic response at low concentrations. While maintaining identical trajectory patterns, co-cultured fibroblasts exposed to 4-aminophenol exhibited an overall lower toxic response than mono-cultured fibroblasts.

### Comparison of GAMM to ANCOVA and ANOVA

**Fig 2:**
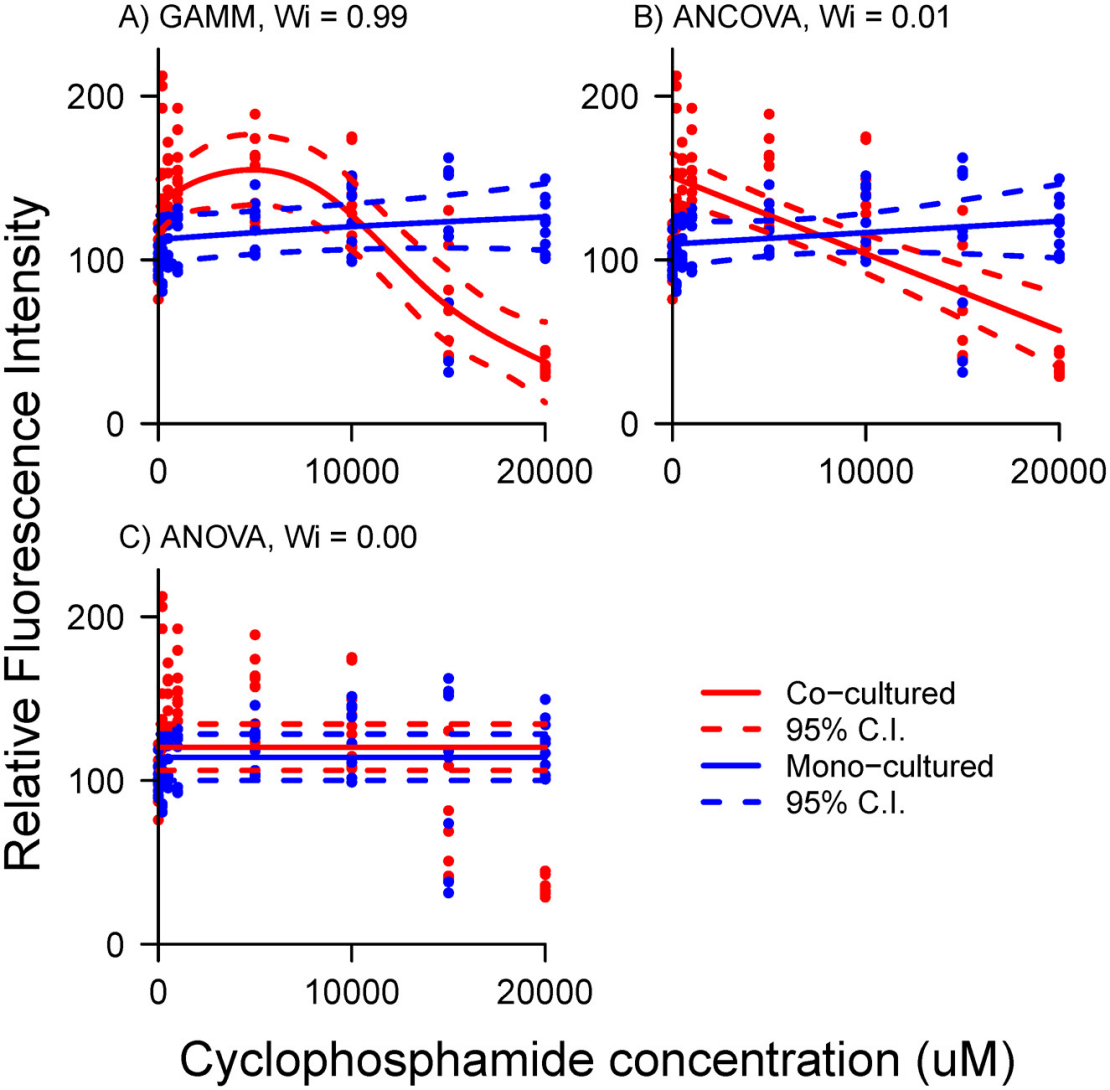
Comparison of A) GAMM, B) ANCOVA and C) ANOVA for cyclophosphamide. ANOCOVA and ANOVA demonstrate insensitivity to the local maximum dose-response indicated by GAMM.

**Fig 3:**
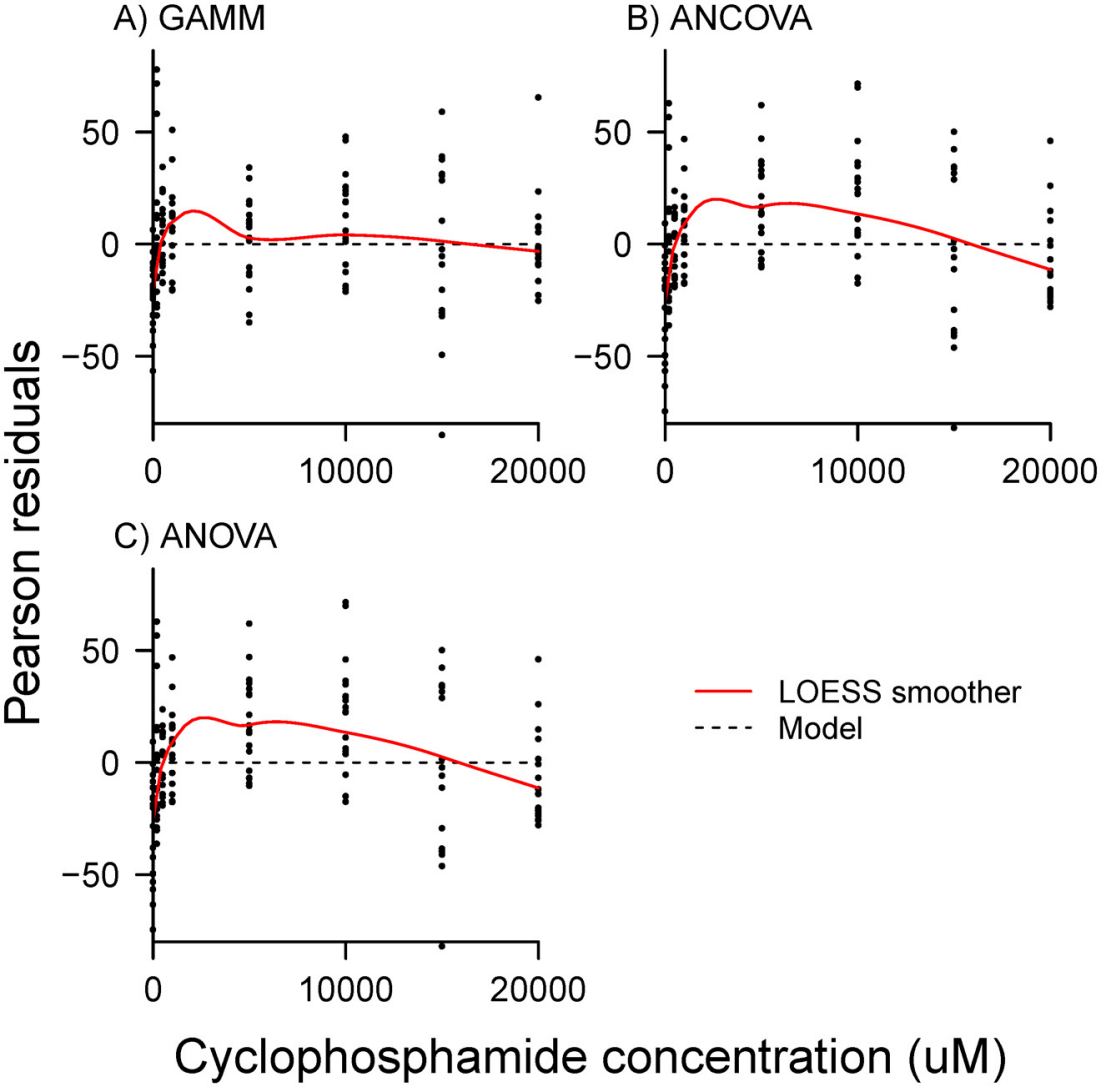
Pearson residuals of A) GAMM, B) ANCOVA and C) ANOVA for cyclophosphamide dose-response. GAMM exhibits a more even distribution of residuals than does ANCOVA or ANOVA (red trace).

AIC comparison of GAMM to mixed-effects ANCOVA and ANOVA, strongly selected the non-linear GAMM for cyclophosphamide response (Fig 2). The standard method of comparing categories with ANOVA rendered virtually no information when compared to ANCOVA and GAMM (*w*_*i*_ = 0.0). While ANCOVA captured the transition from compensatory to toxic response with increasing concentration (Fig 2b), it still had very low information content when compared to the GAMM (*w*_*i*_ = 0.004), due to non-linearity in the response. A comparison of residuals (Fig 3) indicated that variance was most uniform in the GAMM, when compared to ANOVA and ANCOVA, which demonstrated a reduction in heteroscedasticity in the non-linear model.

## Discussion

Selected models allow us to make clear conclusions about dose-response and its interaction with the *in vitro* environment (co-culture vs. mono-culture) in those cases where almost all of the AIC weight is attributed to the preferred model (*w*_*i*_ ≈ 1: Tables 1 & 2). Such clearly preferred models were the case with 4-aminophenol, and with cyclophosphamide exposures, but model-selection for paroxon and ticlopedine dose-response was less clear. While paroxon, and ticlopedine had models where AIC weights were higher than all other models, some support for competing models of lesser preference remained.

The term for co-culture indicated that hepatocytes affected the overall level of response to 4-aminophenol, as would occur when toxicity is reduced by hepatic metabolism. In this case, the shape of the dose-response curves were the same in co and mono-cultured environments, but fluorescence was uniformly more suppressed in mono-culture (Fig 1A)

Interactions with smoothed terms in the case of cyclophosphamide demonstrated a complex response to hepatic metabolites by fibroblasts in co-culture. Examination of the cyclophosphamide dose-response trajectory (Fig 1B), showed that fibroblast fluorescence was enhanced at low doses in the presence of hepatocytes, and then suppressed strongly at high doses (red trace). This suggests that a threshold, or compensatory process such as hormesis occurs, but only when hepatocytes are present. Such effects are obscured unless non-linear models such as GAMMs are used to describe dose-response. Application of cyclophosphamide to fibroblasts produced little effect when hepatocytes were not present (blue trace), so toxicity was attributed to hepatic metabolites. The interaction terms were manifested in the divergent curvature of these two traces.

**Fig 4:**
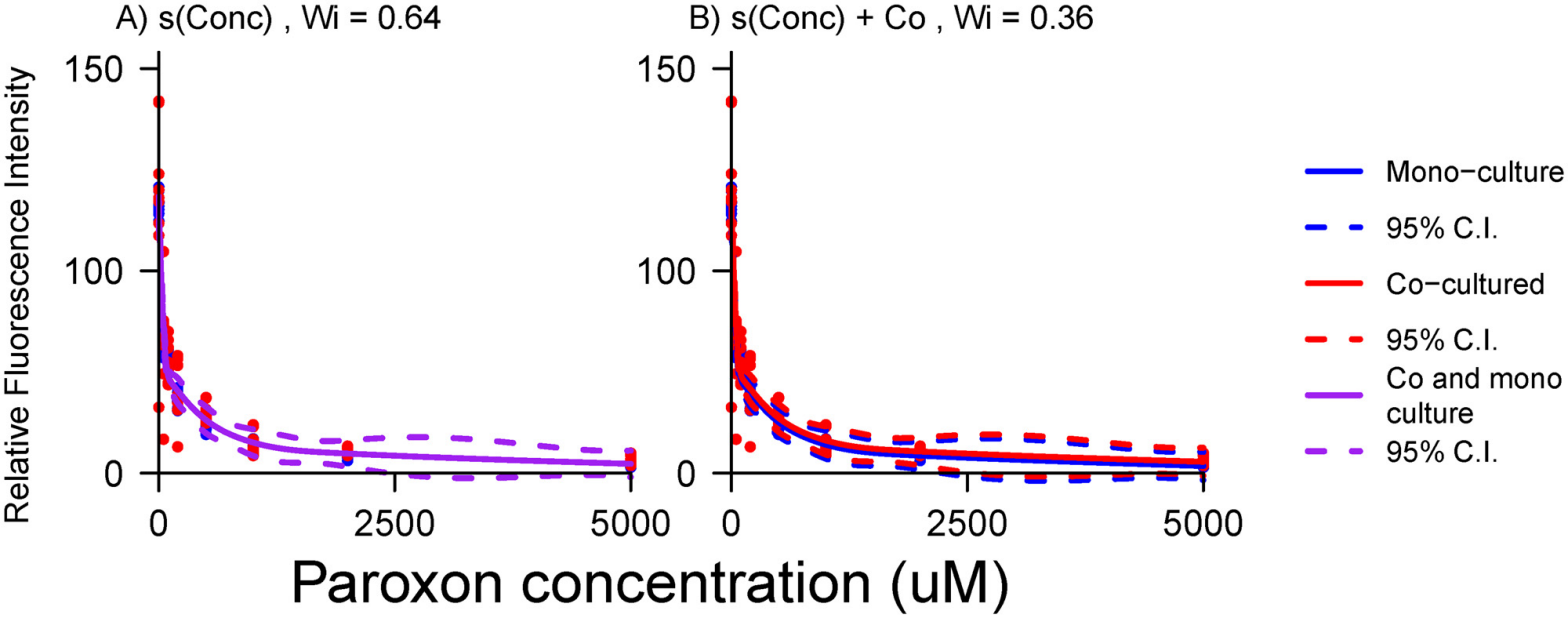
Graphic comparison of alternative models for paroxon dose-response. The AIC preferred model (A, *W*_*i*_ = 0.64) fails to distinguish mono from co-cultured response (purple trace), while the less-preferred, yet still competitive model (B, *W*_*i*_ = 0.36) exhibits a modest difference in intercepts (red vs. blue trace).

While the preferred model for paroxon dose-response indicated no difference between co- and mono-cultured fibroblasts, some lesser support for a competing model with a term for co-culture remained (Table 2). Graphical comparison (Fig 4) exhibited barely perceptible differences between the two competing models, which explained the difficulty in selecting between them with AIC.

**Fig 5:**
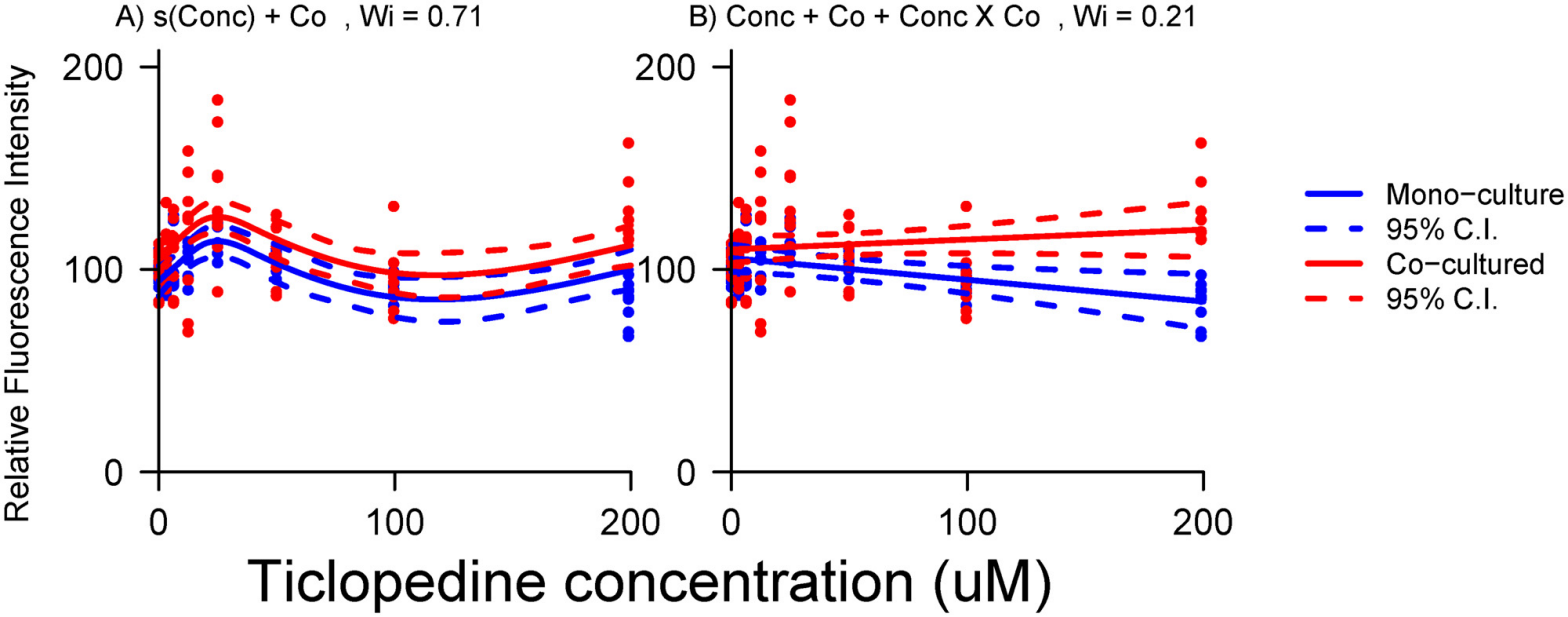
Graphic comparison of alternative models for ticlopedine dose-response. The AIC preferred model (A, *W*_*i*_ = 0.71) exhibits a non-linear response for both mono and co-cultured fibroblasts, while the less-preferred, yet still competitive model (B, *W*_*i*_ = 0.21) exhibits a linear response.

A model with smoothed terms and linear interaction for co-culture was preferred for ticlopedine dose-response, but a competing linear model with interaction term retained some support (Table 1). Graphical comparison of the competing models (Fig 5) showed that, while there are differences between the competing models, curvature in the model with smoothed terms (Fig 5A) is only strong at low doses. The linear model (Fig 5B) did reveal an interesting interaction, showing diverging suppression of fluorescence between mono and co-cultured fibroblasts. However, careful inspection of the more informative non-linear model indicates this interaction is the result of an effect which occurs mostly at low doses, an effect that can’t be revealed by the linear model.

A model-based approach allows formal inference, based on hypothesis tests or information theoretic methods such as AIC, so has advantages over less-formal methods such as bar-plots and t-tests. Model interaction terms show when a difference in dose-response between treatment categories, in our case mono vs. co-culture, can be inferred from data. GAMMs have several distinct advantages over traditional linear models, such as the probit-linked generalized linear models that are familiar to toxicologists, when modeling *in-vitro* systems. GAMMs properly accommodate departures from independence that would otherwise result in psuedoreplication, common within clinical and *in vitro* experimental designs. GAMMs allow changes in trend of response with increasing dose, resulting in a dose-response trajectory. Differences in trajectory between treatment categories can be inferred by interaction terms. Data determine the shape and modality of the curve with GAMMs, so curves are not confined to pre-determined forms. Local maxima apparent in the GAMM may reveal threshold effects or hormesis.

## Supporting Information

S1 Dataset (DOCX)
