# Supplementary material for: Generalized Additive Mixed-Models for Pharmacology Using Integrated Discrete Multiple Organ Co-Culture
Source: PLoS One. 2016 Apr 25;11(4):e0152985. doi: 10.1371/journal.pone.0152985 (PMC4844122; doi:10.1371/journal.pone.0152985)
Supplement: S1 Dataset — (DOCX) [file pone.0152985.s001.docx]

| 4AP | | | |
| --- | --- | --- | --- |
| Relative fluorescence | Concentration | Cell | Co |
| 105.82 | 0 | 13T31 | 0 |
| 110.93 | 2 | 13T32 | 0 |
| 114.52 | 5 | 13T33 | 0 |
| 120.91 | 10 | 13T34 | 0 |
| 27.56 | 50 | 13T35 | 0 |
| 24.59 | 100 | 13T36 | 0 |
| 23.37 | 200 | 13T37 | 0 |
| 12.37 | 500 | 13T38 | 0 |
| 98.67 | 0 | 13T31 | 0 |
| 106.37 | 2 | 13T32 | 0 |
| 119.74 | 5 | 13T33 | 0 |
| 129.84 | 10 | 13T34 | 0 |
| 26.34 | 50 | 13T35 | 0 |
| 22.59 | 100 | 13T36 | 0 |
| 22.99 | 200 | 13T37 | 0 |
| 12.96 | 500 | 13T38 | 0 |
| 95.52 | 0 | 13T31 | 0 |
| 106.03 | 2 | 13T32 | 0 |
| 117.52 | 5 | 13T33 | 0 |
| 114.82 | 10 | 13T34 | 0 |
| 26.32 | 50 | 13T35 | 0 |
| 21.80 | 100 | 13T36 | 0 |
| 22.14 | 200 | 13T37 | 0 |
| 11.38 | 500 | 13T38 | 0 |
| 97.31 | 0 | 23T31 | 0 |
| 107.48 | 2 | 23T32 | 0 |
| 113.66 | 5 | 23T33 | 0 |
| 112.37 | 10 | 23T34 | 0 |
| 21.69 | 50 | 23T35 | 0 |
| 18.28 | 100 | 23T36 | 0 |
| 19.80 | 200 | 23T37 | 0 |
| 10.23 | 500 | 23T38 | 0 |
| 103.15 | 0 | 23T31 | 0 |
| 105.88 | 2 | 23T32 | 0 |
| 111.10 | 5 | 23T33 | 0 |
| 111.75 | 10 | 23T34 | 0 |
| 20.61 | 50 | 23T35 | 0 |
| 19.06 | 100 | 23T36 | 0 |
| 18.27 | 200 | 23T37 | 0 |
| 10.51 | 500 | 23T38 | 0 |
| 99.54 | 0 | 23T31 | 0 |
| 100.58 | 2 | 23T32 | 0 |
| 99.54 | 5 | 23T33 | 0 |
| 108.49 | 10 | 23T34 | 0 |
| 19.96 | 50 | 23T35 | 0 |
| 16.66 | 100 | 23T36 | 0 |
| 20.98 | 200 | 23T37 | 0 |
| 10.21 | 500 | 23T38 | 0 |
| 100.77 | 0 | 33T31 | 0 |
| 104.40 | 2 | 33T32 | 0 |
| 101.43 | 5 | 33T33 | 0 |
| 102.53 | 10 | 33T34 | 0 |
| 19.28 | 50 | 33T35 | 0 |
| 16.41 | 100 | 33T36 | 0 |
| 19.31 | 200 | 33T37 | 0 |
| 10.86 | 500 | 33T38 | 0 |
| 100.82 | 0 | 33T31 | 0 |
| 113.01 | 2 | 33T32 | 0 |
| 103.78 | 5 | 33T33 | 0 |
| 108.78 | 10 | 33T34 | 0 |
| 19.45 | 50 | 33T35 | 0 |
| 16.29 | 100 | 33T36 | 0 |
| 19.00 | 200 | 33T37 | 0 |
| 11.06 | 500 | 33T38 | 0 |
| 98.41 | 0 | 33T31 | 0 |
| 102.38 | 2 | 33T32 | 0 |
| 97.57 | 5 | 33T33 | 0 |
| 100.53 | 10 | 33T34 | 0 |
| 18.04 | 50 | 33T35 | 0 |
| 16.99 | 100 | 33T36 | 0 |
| 18.14 | 200 | 33T37 | 0 |
| 11.14 | 500 | 33T38 | 0 |
| 96.73 | 0 | 1Co1 | 1 |
| 110.97 | 2 | 1Co2 | 1 |
| 116.80 | 5 | 1Co3 | 1 |
| 117.04 | 10 | 1Co4 | 1 |
| 84.65 | 50 | 1Co5 | 1 |
| 72.48 | 100 | 1Co6 | 1 |
| 71.37 | 200 | 1Co7 | 1 |
| 24.35 | 500 | 1Co8 | 1 |
| 108.18 | 0 | 1Co1 | 1 |
| 113.71 | 2 | 1Co2 | 1 |
| 131.42 | 5 | 1Co3 | 1 |
| 124.75 | 10 | 1Co4 | 1 |
| 62.89 | 50 | 1Co5 | 1 |
| 52.95 | 100 | 1Co6 | 1 |
| 72.96 | 200 | 1Co7 | 1 |
| 24.08 | 500 | 1Co8 | 1 |
| 95.09 | 0 | 1Co1 | 1 |
| 95.76 | 2 | 1Co2 | 1 |
| 116.52 | 5 | 1Co3 | 1 |
| 122.90 | 10 | 1Co4 | 1 |
| 78.07 | 50 | 1Co5 | 1 |
| 78.65 | 100 | 1Co6 | 1 |
| 75.06 | 200 | 1Co7 | 1 |
| 22.77 | 500 | 1Co8 | 1 |
| 95.07 | 0 | 2Co1 | 1 |
| 120.40 | 2 | 2Co2 | 1 |
| 112.07 | 5 | 2Co3 | 1 |
| 112.22 | 10 | 2Co4 | 1 |
| 76.04 | 50 | 2Co5 | 1 |
| 59.81 | 100 | 2Co6 | 1 |
| 67.00 | 200 | 2Co7 | 1 |
| 26.77 | 500 | 2Co8 | 1 |
| 104.27 | 0 | 2Co1 | 1 |
| 118.32 | 2 | 2Co2 | 1 |
| 137.21 | 5 | 2Co3 | 1 |
| 141.49 | 10 | 2Co4 | 1 |
| 83.04 | 50 | 2Co5 | 1 |
| 61.15 | 100 | 2Co6 | 1 |
| 88.60 | 200 | 2Co7 | 1 |
| 24.76 | 500 | 2Co8 | 1 |
| 100.66 | 0 | 2Co1 | 1 |
| 104.63 | 2 | 2Co2 | 1 |
| 117.96 | 5 | 2Co3 | 1 |
| 138.32 | 10 | 2Co4 | 1 |
| 88.62 | 50 | 2Co5 | 1 |
| 78.16 | 100 | 2Co6 | 1 |
| 72.74 | 200 | 2Co7 | 1 |
| 26.29 | 500 | 2Co8 | 1 |
| 102.12 | 0 | 3Co1 | 1 |
| 88.26 | 2 | 3Co2 | 1 |
| 102.85 | 5 | 3Co3 | 1 |
| 110.22 | 10 | 3Co4 | 1 |
| 74.11 | 50 | 3Co5 | 1 |
| 62.84 | 100 | 3Co6 | 1 |
| 54.58 | 200 | 3Co7 | 1 |
| 21.05 | 500 | 3Co8 | 1 |
| 103.99 | 0 | 3Co1 | 1 |
| 107.31 | 2 | 3Co2 | 1 |
| 120.97 | 5 | 3Co3 | 1 |
| 130.25 | 10 | 3Co4 | 1 |
| 81.80 | 50 | 3Co5 | 1 |
| 46.12 | 100 | 3Co6 | 1 |
| 43.02 | 200 | 3Co7 | 1 |
| 24.26 | 500 | 3Co8 | 1 |
| 93.89 | 0 | 3Co1 | 1 |
| 87.84 | 2 | 3Co2 | 1 |
| 105.61 | 5 | 3Co3 | 1 |
| 109.80 | 10 | 3Co4 | 1 |
| 97.61 | 50 | 3Co5 | 1 |
| 56.35 | 100 | 3Co6 | 1 |
| 82.04 | 200 | 3Co7 | 1 |
| 21.90 | 500 | 3Co8 | 1 |

| CPA1 | | | |
| --- | --- | --- | --- |
| Relative fluorescence | Concentration | Cell | Co |
| 94.13 | 0 | 13T31 | 0 |
| 84.43 | 200 | 13T32 | 0 |
| 95.59 | 500 | 13T33 | 0 |
| 93.21 | 1000 | 13T34 | 0 |
| 106.35 | 5000 | 13T35 | 0 |
| 111.22 | 10000 | 13T36 | 0 |
| 31.52 | 15000 | 13T37 | 0 |
| 122.97 | 20000 | 13T38 | 0 |
| 90.94 | 0 | 13T31 | 0 |
| 80.83 | 200 | 13T32 | 0 |
| 100 | 500 | 13T33 | 0 |
| 96.1 | 1000 | 13T34 | 0 |
| 102.82 | 5000 | 13T35 | 0 |
| 101.67 | 10000 | 13T36 | 0 |
| 73.99 | 15000 | 13T37 | 0 |
| 116.84 | 20000 | 13T38 | 0 |
| 89.51 | 0 | 13T31 | 0 |
| 85.91 | 200 | 13T32 | 0 |
| 95.89 | 500 | 13T33 | 0 |
| 92.7 | 1000 | 13T34 | 0 |
| 103.85 | 5000 | 13T35 | 0 |
| 99.15 | 10000 | 13T36 | 0 |
| 117.99 | 15000 | 13T37 | 0 |
| 100.96 | 20000 | 13T38 | 0 |
| 93.65 | 0 | 23T31 | 0 |
| 111.31 | 200 | 23T32 | 0 |
| 113.27 | 500 | 23T33 | 0 |
| 125.99 | 1000 | 23T34 | 0 |
| 134.46 | 5000 | 23T35 | 0 |
| 139.01 | 10000 | 23T36 | 0 |
| 114.28 | 15000 | 23T37 | 0 |
| 138.43 | 20000 | 23T38 | 0 |
| 104.16 | 0 | 23T31 | 0 |
| 106.5 | 200 | 23T32 | 0 |
| 121.37 | 500 | 23T33 | 0 |
| 131.56 | 1000 | 23T34 | 0 |
| 127.29 | 5000 | 23T35 | 0 |
| 122.95 | 10000 | 23T36 | 0 |
| 154.71 | 15000 | 23T37 | 0 |
| 125.24 | 20000 | 23T38 | 0 |
| 101.18 | 0 | 23T31 | 0 |
| 98.18 | 200 | 23T32 | 0 |
| 103.08 | 500 | 23T33 | 0 |
| 127.24 | 1000 | 23T34 | 0 |
| 126.38 | 5000 | 23T35 | 0 |
| 139.34 | 10000 | 23T36 | 0 |
| 38.23 | 15000 | 23T37 | 0 |
| 109.75 | 20000 | 23T38 | 0 |
| 109.06 | 0 | 33T31 | 0 |
| 125.55 | 200 | 33T32 | 0 |
| 123.6 | 500 | 33T33 | 0 |
| 125.61 | 1000 | 33T34 | 0 |
| 118.13 | 5000 | 33T35 | 0 |
| 145.92 | 10000 | 33T36 | 0 |
| 151.78 | 15000 | 33T37 | 0 |
| 149.68 | 20000 | 33T38 | 0 |
| 98.46 | 0 | 33T31 | 0 |
| 112.14 | 200 | 33T32 | 0 |
| 123.2 | 500 | 33T33 | 0 |
| 120.78 | 1000 | 33T34 | 0 |
| 129.71 | 5000 | 33T35 | 0 |
| 151.47 | 10000 | 33T36 | 0 |
| 153.96 | 15000 | 33T37 | 0 |
| 103.48 | 20000 | 33T38 | 0 |
| 118.91 | 0 | 33T31 | 0 |
| 124.19 | 200 | 33T32 | 0 |
| 126.47 | 500 | 33T33 | 0 |
| 127.09 | 1000 | 33T34 | 0 |
| 146.08 | 5000 | 33T35 | 0 |
| 144.34 | 10000 | 33T36 | 0 |
| 162.47 | 15000 | 33T37 | 0 |
| 134.15 | 20000 | 33T38 | 0 |
| 97.35 | 0 | 1Co1 | 1 |
| 131.15 | 200 | 1Co2 | 1 |
| 130.33 | 500 | 1Co3 | 1 |
| 136.46 | 1000 | 1Co4 | 1 |
| 120.08 | 5000 | 1Co5 | 1 |
| 107.54 | 10000 | 1Co6 | 1 |
| 40.78 | 15000 | 1Co7 | 1 |
| 33.12 | 20000 | 1Co8 | 1 |
| 100.9 | 0 | 1Co1 | 1 |
| 119.52 | 200 | 1Co2 | 1 |
| 132.8 | 500 | 1Co3 | 1 |
| 149.39 | 1000 | 1Co4 | 1 |
| 134.81 | 5000 | 1Co5 | 1 |
| 114.91 | 10000 | 1Co6 | 1 |
| 69.08 | 15000 | 1Co7 | 1 |
| 36.03 | 20000 | 1Co8 | 1 |
| 94.03 | 0 | 1Co1 | 1 |
| 113.45 | 200 | 1Co2 | 1 |
| 129.12 | 500 | 1Co3 | 1 |
| 141.28 | 1000 | 1Co4 | 1 |
| 123.49 | 5000 | 1Co5 | 1 |
| 128.42 | 10000 | 1Co6 | 1 |
| 109.1 | 15000 | 1Co7 | 1 |
| 102.9 | 20000 | 1Co8 | 1 |
| 87.28 | 0 | 2Co1 | 1 |
| 192.81 | 200 | 2Co2 | 1 |
| 160.85 | 500 | 2Co3 | 1 |
| 147.59 | 1000 | 2Co4 | 1 |
| 157.82 | 5000 | 2Co5 | 1 |
| 140.2 | 10000 | 2Co6 | 1 |
| 81.8 | 15000 | 2Co7 | 1 |
| 31 | 20000 | 2Co8 | 1 |
| 101.14 | 0 | 2Co1 | 1 |
| 206.32 | 200 | 2Co2 | 1 |
| 171.99 | 500 | 2Co3 | 1 |
| 154.27 | 1000 | 2Co4 | 1 |
| 164.12 | 5000 | 2Co5 | 1 |
| 149.71 | 10000 | 2Co6 | 1 |
| 50.98 | 15000 | 2Co7 | 1 |
| 31.48 | 20000 | 2Co8 | 1 |
| 76.08 | 0 | 2Co1 | 1 |
| 212.55 | 200 | 2Co2 | 1 |
| 161.47 | 500 | 2Co3 | 1 |
| 179.66 | 1000 | 2Co4 | 1 |
| 174.23 | 5000 | 2Co5 | 1 |
| 173.61 | 10000 | 2Co6 | 1 |
| 130.41 | 15000 | 2Co7 | 1 |
| 44.97 | 20000 | 2Co8 | 1 |
| 122.27 | 0 | 3Co1 | 1 |
| 128.84 | 200 | 3Co2 | 1 |
| 162.08 | 500 | 3Co3 | 1 |
| 154.93 | 1000 | 3Co4 | 1 |
| 157.3 | 5000 | 3Co5 | 1 |
| 108.71 | 10000 | 3Co6 | 1 |
| 41.9 | 15000 | 3Co7 | 1 |
| 35.27 | 20000 | 3Co8 | 1 |
| 108.34 | 0 | 3Co1 | 1 |
| 153.2 | 200 | 3Co2 | 1 |
| 142.78 | 500 | 3Co3 | 1 |
| 192.81 | 1000 | 3Co4 | 1 |
| 162.55 | 5000 | 3Co5 | 1 |
| 133.35 | 10000 | 3Co6 | 1 |
| 40.55 | 15000 | 3Co7 | 1 |
| 28.88 | 20000 | 3Co8 | 1 |
| 112.62 | 0 | 3Co1 | 1 |
| 137.56 | 200 | 3Co2 | 1 |
| 153.15 | 500 | 3Co3 | 1 |
| 162.73 | 1000 | 3Co4 | 1 |
| 189.15 | 5000 | 3Co5 | 1 |
| 175.27 | 10000 | 3Co6 | 1 |
| 39.24 | 15000 | 3Co7 | 1 |
| 42.78 | 20000 | 3Co8 | 1 |

| Paroxon | | | |
| --- | --- | --- | --- |
| Relative fluorescence | Concentration | Cell | Co |
| 93.48 | 0 | 13T31 | 0 |
| 42.87 | 50 | 13T32 | 0 |
| 36.92 | 100 | 13T33 | 0 |
| 23.27 | 200 | 13T34 | 0 |
| 14.45 | 500 | 13T35 | 0 |
| 9.36 | 1000 | 13T36 | 0 |
| 4.75 | 2000 | 13T37 | 0 |
| 2.48 | 5000 | 13T38 | 0 |
| 106.16 | 0 | 13T31 | 0 |
| 47.98 | 50 | 13T32 | 0 |
| 40.48 | 100 | 13T33 | 0 |
| 26.50 | 200 | 13T34 | 0 |
| 16.38 | 500 | 13T35 | 0 |
| 10.67 | 1000 | 13T36 | 0 |
| 5.99 | 2000 | 13T37 | 0 |
| 2.35 | 5000 | 13T38 | 0 |
| 100.36 | 0 | 13T31 | 0 |
| 46.55 | 50 | 13T32 | 0 |
| 40.71 | 100 | 13T33 | 0 |
| 26.93 | 200 | 13T34 | 0 |
| 17.88 | 500 | 13T35 | 0 |
| 11.78 | 1000 | 13T36 | 0 |
| 5.99 | 2000 | 13T37 | 0 |
| 2.78 | 5000 | 13T38 | 0 |
| 96.23 | 0 | 23T31 | 0 |
| 50.37 | 50 | 23T32 | 0 |
| 39.60 | 100 | 23T33 | 0 |
| 28.50 | 200 | 23T34 | 0 |
| 19.91 | 500 | 23T35 | 0 |
| 10.79 | 1000 | 23T36 | 0 |
| 6.83 | 2000 | 23T37 | 0 |
| 2.43 | 5000 | 23T38 | 0 |
| 104.82 | 0 | 23T31 | 0 |
| 54.61 | 50 | 23T32 | 0 |
| 37.80 | 100 | 23T33 | 0 |
| 27.88 | 200 | 23T34 | 0 |
| 16.89 | 500 | 23T35 | 0 |
| 10.76 | 1000 | 23T36 | 0 |
| 7.42 | 2000 | 23T37 | 0 |
| 2.38 | 5000 | 23T38 | 0 |
| 98.95 | 0 | 23T31 | 0 |
| 50.87 | 50 | 23T32 | 0 |
| 38.28 | 100 | 23T33 | 0 |
| 24.21 | 200 | 23T34 | 0 |
| 16.53 | 500 | 23T35 | 0 |
| 10.23 | 1000 | 23T36 | 0 |
| 7.46 | 2000 | 23T37 | 0 |
| 2.32 | 5000 | 23T38 | 0 |
| 100.13 | 0 | 33T31 | 0 |
| 49.13 | 50 | 33T32 | 0 |
| 46.03 | 100 | 33T33 | 0 |
| 31.59 | 200 | 33T34 | 0 |
| 17.64 | 500 | 33T35 | 0 |
| 11.99 | 1000 | 33T36 | 0 |
| 6.50 | 2000 | 33T37 | 0 |
| 2.34 | 5000 | 33T38 | 0 |
| 97.74 | 0 | 33T31 | 0 |
| 47.48 | 50 | 33T32 | 0 |
| 43.10 | 100 | 33T33 | 0 |
| 30.19 | 200 | 33T34 | 0 |
| 18.67 | 500 | 33T35 | 0 |
| 12.60 | 1000 | 33T36 | 0 |
| 6.80 | 2000 | 33T37 | 0 |
| 2.40 | 5000 | 33T38 | 0 |
| 102.13 | 0 | 33T31 | 0 |
| 49.98 | 50 | 33T32 | 0 |
| 41.88 | 100 | 33T33 | 0 |
| 31.74 | 200 | 33T34 | 0 |
| 18.92 | 500 | 33T35 | 0 |
| 12.80 | 1000 | 33T36 | 0 |
| 6.78 | 2000 | 33T37 | 0 |
| 2.55 | 5000 | 33T38 | 0 |
| 111.05 | 0 | 1Co1 | 1 |
| 56.63 | 50 | 1Co2 | 1 |
| 46.63 | 100 | 1Co3 | 1 |
| 29.13 | 200 | 1Co4 | 1 |
| 18.65 | 500 | 1Co5 | 1 |
| 12.59 | 1000 | 1Co6 | 1 |
| 7.29 | 2000 | 1Co7 | 1 |
| 2.97 | 5000 | 1Co8 | 1 |
| 100.75 | 0 | 1Co1 | 1 |
| 49.92 | 50 | 1Co2 | 1 |
| 46.49 | 100 | 1Co3 | 1 |
| 26.20 | 200 | 1Co4 | 1 |
| 21.47 | 500 | 1Co5 | 1 |
| 6.62 | 1000 | 1Co6 | 1 |
| 7.42 | 2000 | 1Co7 | 1 |
| 2.90 | 5000 | 1Co8 | 1 |
| 88.20 | 0 | 1Co1 | 1 |
| 48.42 | 50 | 1Co2 | 1 |
| 33.23 | 100 | 1Co3 | 1 |
| 28.63 | 200 | 1Co4 | 1 |
| 23.75 | 500 | 1Co5 | 1 |
| 11.12 | 1000 | 1Co6 | 1 |
| 8.05 | 2000 | 1Co7 | 1 |
| 3.46 | 5000 | 1Co8 | 1 |
| 138.03 | 0 | 2Co1 | 1 |
| 82.22 | 50 | 2Co2 | 1 |
| 32.88 | 100 | 2Co3 | 1 |
| 42.52 | 200 | 2Co4 | 1 |
| 28.14 | 500 | 2Co5 | 1 |
| 8.20 | 1000 | 2Co6 | 1 |
| 9.36 | 2000 | 2Co7 | 1 |
| 7.52 | 5000 | 2Co8 | 1 |
| 24.50 | 0 | 2Co1 | 1 |
| 12.66 | 50 | 2Co2 | 1 |
| 52.64 | 100 | 2Co3 | 1 |
| 43.67 | 200 | 2Co4 | 1 |
| 27.87 | 500 | 2Co5 | 1 |
| 18.04 | 1000 | 2Co6 | 1 |
| 10.11 | 2000 | 2Co7 | 1 |
| 5.27 | 5000 | 2Co8 | 1 |
| 137.47 | 0 | 2Co1 | 1 |
| 47.60 | 50 | 2Co2 | 1 |
| 49.46 | 100 | 2Co3 | 1 |
| 40.05 | 200 | 2Co4 | 1 |
| 23.75 | 500 | 2Co5 | 1 |
| 17.30 | 1000 | 2Co6 | 1 |
| 9.21 | 2000 | 2Co7 | 1 |
| 6.60 | 5000 | 2Co8 | 1 |
| 102.17 | 0 | 3Co1 | 1 |
| 55.53 | 50 | 3Co2 | 1 |
| 33.66 | 100 | 3Co3 | 1 |
| 9.86 | 200 | 3Co4 | 1 |
| 19.14 | 500 | 3Co5 | 1 |
| 7.37 | 1000 | 3Co6 | 1 |
| 6.60 | 2000 | 3Co7 | 1 |
| 3.96 | 5000 | 3Co8 | 1 |
| 105.09 | 0 | 3Co1 | 1 |
| 50.42 | 50 | 3Co2 | 1 |
| 38.24 | 100 | 3Co3 | 1 |
| 28.95 | 200 | 3Co4 | 1 |
| 20.94 | 500 | 3Co5 | 1 |
| 9.41 | 1000 | 3Co6 | 1 |
| 7.57 | 2000 | 3Co7 | 1 |
| 3.61 | 5000 | 3Co8 | 1 |
| 92.74 | 0 | 3Co1 | 1 |
| 36.91 | 50 | 3Co2 | 1 |
| 41.22 | 100 | 3Co3 | 1 |
| 23.56 | 200 | 3Co4 | 1 |
| 20.09 | 500 | 3Co5 | 1 |
| 11.17 | 1000 | 3Co6 | 1 |
| 7.14 | 2000 | 3Co7 | 1 |
| 3.90 | 5000 | 3Co8 | 1 |

| Ticlopedine | | | |
| --- | --- | --- | --- |
| Relative fluorescence | Concentration | Cell | Co |
| 96.01 | 0 | 13T31 | 0 |
| 97.35 | 3.1 | 13T32 | 0 |
| 124.14 | 6.2 | 13T33 | 0 |
| 113.74 | 12.4 | 13T34 | 0 |
| 121.11 | 24.9 | 13T35 | 0 |
| 109.21 | 49.8 | 13T36 | 0 |
| 90.52 | 99.5 | 13T37 | 0 |
| 92.64 | 199 | 13T38 | 0 |
| 102.42 | 0 | 13T31 | 0 |
| 105.87 | 3.1 | 13T32 | 0 |
| 126.89 | 6.2 | 13T33 | 0 |
| 111.48 | 12.4 | 13T34 | 0 |
| 124.15 | 24.9 | 13T35 | 0 |
| 104.76 | 49.8 | 13T36 | 0 |
| 93.83 | 99.5 | 13T37 | 0 |
| 89.41 | 199 | 13T38 | 0 |
| 101.57 | 0 | 13T31 | 0 |
| 104.55 | 3.1 | 13T32 | 0 |
| 114.34 | 6.2 | 13T33 | 0 |
| 108.76 | 12.4 | 13T34 | 0 |
| 125.42 | 24.9 | 13T35 | 0 |
| 104.68 | 49.8 | 13T36 | 0 |
| 99.22 | 99.5 | 13T37 | 0 |
| 79.02 | 199 | 13T38 | 0 |
| 93.97 | 0 | 23T31 | 0 |
| 89.19 | 3.1 | 23T32 | 0 |
| 93.22 | 6.2 | 23T33 | 0 |
| 90.04 | 12.4 | 23T34 | 0 |
| 108.10 | 24.9 | 23T35 | 0 |
| 95.14 | 49.8 | 23T36 | 0 |
| 82.25 | 99.5 | 23T37 | 0 |
| 67.01 | 199 | 23T38 | 0 |
| 102.19 | 0 | 23T31 | 0 |
| 98.72 | 3.1 | 23T32 | 0 |
| 95.07 | 6.2 | 23T33 | 0 |
| 95.02 | 12.4 | 23T34 | 0 |
| 103.44 | 24.9 | 23T35 | 0 |
| 99.30 | 49.8 | 23T36 | 0 |
| 82.34 | 99.5 | 23T37 | 0 |
| 69.29 | 199 | 23T38 | 0 |
| 103.84 | 0 | 23T31 | 0 |
| 87.29 | 3.1 | 23T32 | 0 |
| 92.02 | 6.2 | 23T33 | 0 |
| 96.84 | 12.4 | 23T34 | 0 |
| 111.50 | 24.9 | 23T35 | 0 |
| 101.40 | 49.8 | 23T36 | 0 |
| 88.32 | 99.5 | 23T37 | 0 |
| 97.39 | 199 | 23T38 | 0 |
| 110.01 | 0 | 33T31 | 0 |
| 104.61 | 3.1 | 33T32 | 0 |
| 94.65 | 6.2 | 33T33 | 0 |
| 108.69 | 12.4 | 33T34 | 0 |
| 123.08 | 24.9 | 33T35 | 0 |
| 103.62 | 49.8 | 33T36 | 0 |
| 92.62 | 99.5 | 33T37 | 0 |
| 86.87 | 199 | 33T38 | 0 |
| 98.65 | 0 | 33T31 | 0 |
| 107.14 | 3.1 | 33T32 | 0 |
| 98.60 | 6.2 | 33T33 | 0 |
| 105.76 | 12.4 | 33T34 | 0 |
| 121.12 | 24.9 | 33T35 | 0 |
| 110.43 | 49.8 | 33T36 | 0 |
| 96.92 | 99.5 | 33T37 | 0 |
| 90.01 | 199 | 33T38 | 0 |
| 91.34 | 0 | 33T31 | 0 |
| 104.15 | 3.1 | 33T32 | 0 |
| 94.32 | 6.2 | 33T33 | 0 |
| 104.76 | 12.4 | 33T34 | 0 |
| 113.30 | 24.9 | 33T35 | 0 |
| 105.98 | 49.8 | 33T36 | 0 |
| 86.69 | 99.5 | 33T37 | 0 |
| 85.45 | 199 | 33T38 | 0 |
| 112.83 | 0 | 1Co1 | 1 |
| 108.50 | 3.1 | 1Co2 | 1 |
| 115.07 | 6.2 | 1Co3 | 1 |
| 126.45 | 12.4 | 1Co4 | 1 |
| 128.82 | 24.9 | 1Co5 | 1 |
| 106.90 | 49.8 | 1Co6 | 1 |
| 89.29 | 99.5 | 1Co7 | 1 |
| 118.77 | 199 | 1Co8 | 1 |
| 83.43 | 0 | 1Co1 | 1 |
| 93.38 | 3.1 | 1Co2 | 1 |
| 84.71 | 6.2 | 1Co3 | 1 |
| 69.32 | 12.4 | 1Co4 | 1 |
| 146.54 | 24.9 | 1Co5 | 1 |
| 121.32 | 49.8 | 1Co6 | 1 |
| 103.39 | 99.5 | 1Co7 | 1 |
| 124.53 | 199 | 1Co8 | 1 |
| 103.75 | 0 | 1Co1 | 1 |
| 102.76 | 3.1 | 1Co2 | 1 |
| 110.82 | 6.2 | 1Co3 | 1 |
| 125.77 | 12.4 | 1Co4 | 1 |
| 89.07 | 24.9 | 1Co5 | 1 |
| 89.20 | 49.8 | 1Co6 | 1 |
| 86.69 | 99.5 | 1Co7 | 1 |
| 118.27 | 199 | 1Co8 | 1 |
| 99.84 | 0 | 2Co1 | 1 |
| 114.78 | 3.1 | 2Co2 | 1 |
| 116.22 | 6.2 | 2Co3 | 1 |
| 133.64 | 12.4 | 2Co4 | 1 |
| 121.79 | 24.9 | 2Co5 | 1 |
| 106.99 | 49.8 | 2Co6 | 1 |
| 79.56 | 99.5 | 2Co7 | 1 |
| 114.90 | 199 | 2Co8 | 1 |
| 103.61 | 0 | 2Co1 | 1 |
| 90.51 | 3.1 | 2Co2 | 1 |
| 113.28 | 6.2 | 2Co3 | 1 |
| 124.69 | 12.4 | 2Co4 | 1 |
| 145.58 | 24.9 | 2Co5 | 1 |
| 127.22 | 49.8 | 2Co6 | 1 |
| 96.42 | 99.5 | 2Co7 | 1 |
| 118.28 | 199 | 2Co8 | 1 |
| 96.54 | 0 | 2Co1 | 1 |
| 106.27 | 3.1 | 2Co2 | 1 |
| 83.51 | 6.2 | 2Co3 | 1 |
| 73.34 | 12.4 | 2Co4 | 1 |
| 111.31 | 24.9 | 2Co5 | 1 |
| 87.12 | 49.8 | 2Co6 | 1 |
| 75.91 | 99.5 | 2Co7 | 1 |
| 114.95 | 199 | 2Co8 | 1 |
| 84.33 | 0 | 3Co1 | 1 |
| 133.15 | 3.1 | 3Co2 | 1 |
| 96.81 | 6.2 | 3Co3 | 1 |
| 148.12 | 12.4 | 3Co4 | 1 |
| 124.29 | 24.9 | 3Co5 | 1 |
| 120.60 | 49.8 | 3Co6 | 1 |
| 93.79 | 99.5 | 3Co7 | 1 |
| 143.35 | 199 | 3Co8 | 1 |
| 109.03 | 0 | 3Co1 | 1 |
| 93.79 | 3.1 | 3Co2 | 1 |
| 129.77 | 6.2 | 3Co3 | 1 |
| 94.88 | 12.4 | 3Co4 | 1 |
| 172.92 | 24.9 | 3Co5 | 1 |
| 101.35 | 49.8 | 3Co6 | 1 |
| 131.25 | 99.5 | 3Co7 | 1 |
| 162.54 | 199 | 3Co8 | 1 |
| 106.64 | 0 | 3Co1 | 1 |
| 117.57 | 3.1 | 3Co2 | 1 |
| 125.39 | 6.2 | 3Co3 | 1 |
| 158.58 | 12.4 | 3Co4 | 1 |
| 183.77 | 24.9 | 3Co5 | 1 |
| 111.50 | 49.8 | 3Co6 | 1 |
| 98.40 | 99.5 | 3Co7 | 1 |
| 128.89 | 199 | 3Co8 | 1 |
